# Consensus on treatment for residents in long-term care facilities: perspectives from relatives and care staff in the PACE cross-sectional study in 6 European countries

**DOI:** 10.1186/s12904-019-0459-9

**Published:** 2019-08-29

**Authors:** M. ten Koppel, H. R. W. Pasman, J. T. van der Steen, H. P. J. van Hout, M. Kylänen, L. Van den Block, T. Smets, L. Deliens, G. Gambassi, K. Froggatt, K. Szczerbińska, B. D. Onwuteaka-Philipsen, PACE, Zeger De Groote, Lara Pivodic, Federica Mammarella, Martina Mercuri, Mariska Oosterveld-Vlug, Agnieszka Pac, Paola Rossi, Ivan Segat, Eleanor Sowerby, Agata Stodolska, Anne Wichmann, Eddy Adang, Paula Andreasen, Harriet Finne-Soveri, Sheila Payne, Danni Collingridge Moore, Violetta Kijowska, Nele Van Den Noortgate, Myrra Vernooij-Dassen

**Affiliations:** 1 Department of Public and Occupational Health, Amsterdam Public Health Research Institute, Expertise Center for Palliative Care, Amsterdam UMC, Vrije Universiteit Amsterdam, Amsterdam, The Netherlands, Van der Boechorststraat 7, 1081 BT Amsterdam, The Netherlands; 2 Department of Public Health and Primary Care, Leiden University Medical Center, Albinusdreef 2, Leiden, The Netherlands; 3 Department of Primary and Community Care, Radboud University Medical Center, Geert Grooteplein Zuid 10, Nijmegen, The Netherlands; 4 Department of General Practice & Elderly Care Medicine, Amsterdam Public Health Research Institute, Amsterdam UMC, Vrije Universiteit Amsterdam, Van der Boechorststraat 7, Amsterdam, The Netherlands; 5 National Institute for Health and Welfare, Mannerheimintie, 166 Helsinki, Finland; 6 End-of-Life Care Research Group, Vrije Universiteit Brussel (VUB) and Ghent University, Laarbeeklaan, 103 Brussels, Belgium; 7 Università Cattolica del Sacro Cuore, Largo Francesco Vito 1, Rome, Italy; 8 International Observatory on End-of-Life Care, Lancaster University, Lancaster, LA1 4YG UK; 9 Unit for Research on Aging Society, Department of Sociology of Medicine, Epidemiology and Preventive Medicine Chair, Faculty of Medicine, Jagiellonian University Medical College, ul. Kopernika 7a, Krakow, Poland

**Keywords:** Cross-sectional studies, End-of-life care, Health communication, Nursing homes

## Abstract

**Background:**

In long-term care facilities often many care providers are involved, which could make it difficult to reach consensus in care. This may harm the relation between care providers and can complicate care. This study aimed to describe and compare in six European countries the degree of consensus among everyone involved in care decisions, from the perspective of relatives and care staff. Another aim was to assess which factors are associated with reporting that full consensus was reached, from the perspective of care staff and relatives.

**Methods:**

In Belgium, England, Finland, Italy, the Netherlands and Poland a random sample of representative long-term care facilities reported all deaths of residents in the previous three months (*n* = 1707). This study included residents about whom care staff (*n* = 1284) and relatives (*n* = 790) indicated in questionnaires the degree of consensus among all involved in the decision or care process. To account for clustering on facility level, Generalized Estimating Equations were conducted to analyse the degree of consensus across countries and factors associated with full consensus.

**Results:**

Relatives indicated full consensus in more than half of the residents in all countries (NL 57.9% - EN 68%), except in Finland (40.7%). Care staff reported full consensus in 59.5% of residents in Finland to 86.1% of residents in England. Relatives more likely reported full consensus when: the resident was more comfortable or talked about treatment preferences, a care provider explained what palliative care is, family-physician communication was well perceived, their relation to the resident was other than child (compared to spouse/partner) or if they lived in Poland or Belgium (compared to Finland). Care staff more often indicated full consensus when they rated a higher comfort level of the resident, or if they lived in Italy, the Netherland, Poland or England (compared to Finland).

**Conclusions:**

In most countries the frequency of full consensus among all involved in care decisions was relatively high. Across countries care staff indicated full consensus more often and no consensus less often than relatives. Advance care planning, comfort and good communication between relatives and care professionals could play a role in achieving full consensus.

**Electronic supplementary material:**

The online version of this article (10.1186/s12904-019-0459-9) contains supplementary material, which is available to authorized users.

## Background

When older people are living in long-term care facilities (LTCFs), both formal and informal caregivers including families are usually involved in making decisions about care and treatments, especially at the end of life [1–5]. In the process of making such decisions, shared decision-making and the documentation of decisions for (future) care in care plans have become increasingly important [6–9]. Physicians, nurses and families usually favour consensus in clinical decision making [10–12]. As for other medical decisions, end-of-life decisions are preference sensitive, and therefore based not only on direct facts but also on personal values and individual perspectives [13, 14]. Eliciting and negotiating preferences and facts, can complicate reaching a consensus in decision making. Not being able to find common grounds could generate a conflict between care professionals and family members and can hinder adequate care giving [15] and a good end of life from the perspective of families [16].

Previous research has documented that attitudes and perspectives towards end-of-life (decisions) can diverge between those involved in the care of older people residing in nursing homes. For example, in USA nursing staff more often reported that death of the resident was expected and cited a higher symptom burden compared to family members [17]. In the Netherlands, physicians, nurses and relatives have been reported to display different attitudes towards the importance of advance directives or hastening death [18]. For instance, compared to physicians, nurses less often agreed that forgoing artificial nutrition and hydration is almost always followed by a peaceful death. And compared to relatives, physicians less often agreed that an advance directive should always be followed. Divergent care goals between family caregivers of community-dwelling older adults and physicians were also documented in the USA and in Canada [19, 20].

While these studies indicate that reaching a consensus could be an issue in the end-of-life care of older people in LTCFs, little is known about how relatives and care staff of long-term care residents actually perceive the degree of consensus regarding decisions on care and treatment that have been made at the end of life. As part of the PACE project [21], we assessed consensus on care and treatment for long-term care residents in a large sample of European LTCFs. More specifically, our aim was to describe the degree of consensus among those involved, from the perspective of relatives and care staff, and to compare such degree of consensus across six European countries. Additionally, we ought to assess which factors were associated with reporting a full consensus, both from the perspective of care staff and of relatives.

## Methods

### Study setting

The present study used data collected in 2015 as part of the “Palliative Care for Older People” (PACE) project, which included a cross-sectional study of deceased residents in LTCFs in Belgium, England, Finland, Italy, the Netherlands and Poland [21]. In each country a representative sample of facilities was obtained with use of proportional stratified random sampling, taking into account region, facility type and bed capacity. In Italy, where no public listing of LTCFs was available, a convenience sample was obtained based on a previously constructed cluster of LTCFs, covering the three macro regional areas of the country and accounting for the facility sizes and types in the country [22]. Each LTCF reported all deaths of residents in or outside the facility in the 3 months preceding participation. A structured questionnaire concerning each deceased resident was distributed in the facility or sent by mail to: the facility administrator; the care staff member most involved in the care of the resident (either a nurse or a care assistant); and a relative who was closely involved. A questionnaire on facility characteristics was also collected from the facility administrator. Questionnaires were accompanied by a letter containing information about the study, which asked for participation and explained that the participant’s data would remain anonymous.

An administrator in the facility listed the care staff and relatives who were most closely involved in the care for the resident and assigned anonymous codes to all questionnaires, assisted by a researcher. This administrator also distributed and mailed the questionnaires and sent out a maximum of two reminders to non-responders. Participants mailed the questionnaires back directly to the research team.

### Ethics

Ethical approval was obtained from the relevant ethics committees in each country. In the Netherlands and Italy a waiver was obtained for the collection of data of deceased residents. All respondents participated on a voluntary basis and their responses remained anonymous, therefore their written responses were taken as valid informed consent.

### Measurements

The degree of consensus among all involved was measured with the following questions, also used in the Dutch End Of Life in Dementia (DEOLD) study [23]:

- *Relatives*: ‘To what degree did all those who were involved in the treatment(s) decision(s) (LTCF staff, family members, others) agree about the best treatment(s)?’ *(full consensus, consensus on major issues, no consensus)*
- *Care staff*: ‘To which degree were those involved in care in agreement (consensus) on care and treatment in the last month of the resident’s life? a) among staff themselves b) among family themselves c) among all those involved *(full consensus, consensus on major issues, no consensus)*

Regarding factors possibly associated with full consensus we considered the following, based on literature (justification for variables displayed in Additional file 1: Table S1):

- *Resident characteristics (questionnaire for administrator, staff and relatives)*: country, length of stay (days), health in the last week of life (0–100, EQ-5DL5) [24, 25], comfort level in the last week of life (14–24, End-of-Life in Dementia – Comfort Assessment in Dying (EOLD-CAD)) [26], diagnosis of dementia, resident spoke with care staff/relative or someone else about their preferences for treatment at the end of life. Both relatives and care staff assessed health, comfort level, dementia, and whether the resident spoke about treatments preferences.
- *Care facility characteristics (questionnaire for administrator)*: organization of multidisciplinary meetings in facility, number of care staff per 10 occupied beds.
- *Relative characteristics (questionnaire for relatives):* relationship to resident, care provider explained what palliative care means, relative did not really understand the resident’s condition, relative expected resident would die (one month before death), relative felt fully involved in all decision making, Family Perception of Physician-Family Communication (FPPFC) scale (0–3) [27].

A forward-backward translation according to the EORTC guidelines [28] was conducted for all measurements for which a validated version was not available in the different languages. These cross-cultural translations of all questionnaires were pilot-tested among all target groups. Based upon the results of the pilot-test, any necessary adjustments were made to the translated questionnaires.

### Sample

**Fig. 1:**
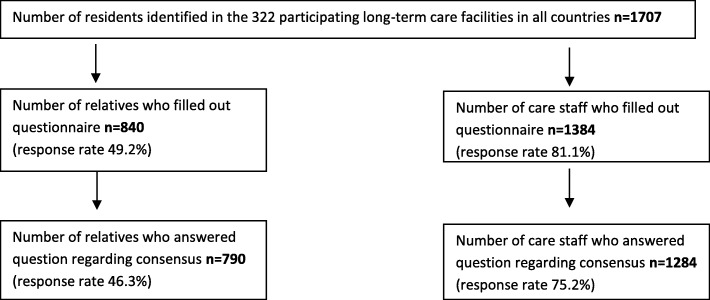
Number of relatives and care staff included in the current study

The PACE project included 1707 residents in 322 facilities. This study used two selection criteria, based on questionnaires filled out by relatives and questionnaires filled out by staff (see Fig. 1), which included:

- 790 residents about whom *relatives* answered the question regarding consensus among all involved, out of 840 residents about whom relatives filled out the questionnaire.
- 1284 residents about whom *care staff* (nurse or care assistant) answered the question regarding consensus among all involved, out of 1384 residents about whom care staff filled out the questionnaire.

### Analyses

Descriptive statistics were reported for resident characteristics and the degree of consensus as indicated by relatives and care staff.

To account for clustering of data in facilities, logistic Generalized Estimating Equations (GEE) with an exchangeable correlation structure was used for all further analyses. To use the degree of consensus as a dependent variable in GEE analyses and prevent non-convergence due to empty cells, response options were dichotomised into: full consensus, no full consensus (combining consensus on major issues and no consensus).

Differences between countries were analysed using dummy variables of country as the independent variable and consensus according to relatives and consensus according to care staff as dependent variables.

Associations between full consensus according to relatives and characteristics of residents, relatives and care facilities were assessed and associations between full consensus according to care staff and characteristics of residents and care facilitieswere assessed. First, univariable analyses were conducted. Next, all factors were included in the multivariable GEE models and manual stepwise backward selection identified the factors most strongly associated (*p*-value <.05) with full consensus. An interaction term with country was added to each independent variable in the final multivariable model, to evaluate possible effect modification by country. Analyses regarding consensus according to relatives included resident characteristics as assessed by relatives, while analyses regarding consensus according to care staff included resident characteristics as assessed by care staff.

Continuous independent variables that did not show a linear relation with the dependent variables, were dichotomised on either the mean or median value. *P*-values < 0.05 were considered statistically significant. All analyses were conducted in SPSS 23 [29].

## Results

### Population

In both samples, just over half of the residents were over 85 years of age, about 2/3 were female and the mean comfort level in the last week of life was about 30 (Table 1). Relatives indicated dementia in 55.4% of residents, compared to 70.5% indicated by care staff, who rated higher levels of health in the last week, compared to relatives (median 20 vs. 10).

**Table 1:** Characteristics of the residents

	Residents about whom *relatives* filled in the question on consensus amongst all involved (*n* = 790)	Residents about whom *care staff* filled in the question on consensus amongst all involved (*n* = 1284)
Resident characteristics		
Age > 85 n (%)	410 (54.8)	686 (55.4)
Sex, female n (%)	520 (68.6)	814 (65.7)
Dementia* n (%)	424 (55.4)	898 (70.5)
Health in last week of life*^a^ median (IQR)	10 (5.0–25.0)	20 (10.0–40.0)
Comfort level during the last week of life*^b^ mean (sd)	29.25 (5.72)	30.61 (5.37)

*As assessed by relative and care staff, respectively

^a^Measured by EQ-5DLD5, higher scores indicate a better health status (0–100)

^b^Measured by EOLD-CAD, higher scores indicate a higher comfort level (14–42)

Missing values care staff: age 46, sex 45, dementia 10, health 51, comfort 67

Missing values relatives: age 42, sex 32, dementia 24, health 35, comfort 87

### Degree of consensus

**Fig. 2:**
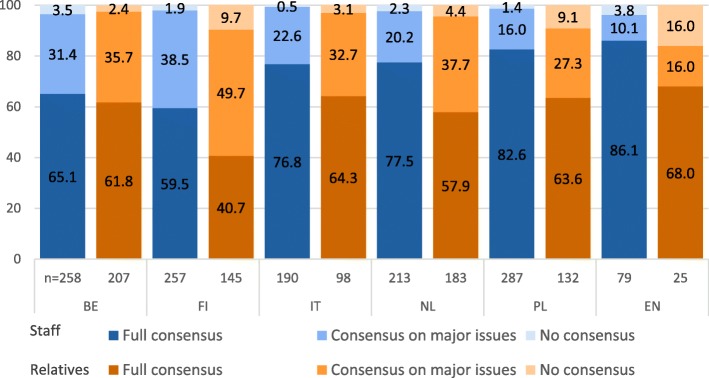
Degree of consensus amongst all involved according to care staff and according to relatives, across different countries. Due to rounding up of decimal places, not all columns add up to 100%

Relatives reported the degree of consensus among all involved in treatment decisions for 790 residents (see Fig. 2). Relatives indicated no consensus was reached in 16% of residents in England, in 9.1 and 9.7% in Poland and Finland respectively and in less than 5% of residents in the other countries. In England and Poland relatives reported consensus on major issues was reached in 16 and 27.3% of residents, while in the other countries this ranged from over 30% up to 49.7% in Finland. The proportion of relatives who indicated full consensus was 40.7% in Finland, which differed significantly from the other countries where full consensus ranged from 57.9% in the Netherlands to 68% in England.

Care staff reported the degree of consensus on care and treatment in the last month of life among all those involved for 1284 residents. According to staff, no consensus was reached in less than 4% of residents in all countries. Care staff reported consensus on major issues was reached in 38.5 and 31.4% of residents in Finland and Belgium respectively, and in less than 25% of residents in the other countries. In Finland and Belgium care staff indicated full consensus significantly less often (59.5 and 65.1% respectively) than in England (86.1%), the Netherlands (77.5%) and Poland (82.6%). Italy (76.8%) also differed significantly from Finland.

**Fig. 3:**
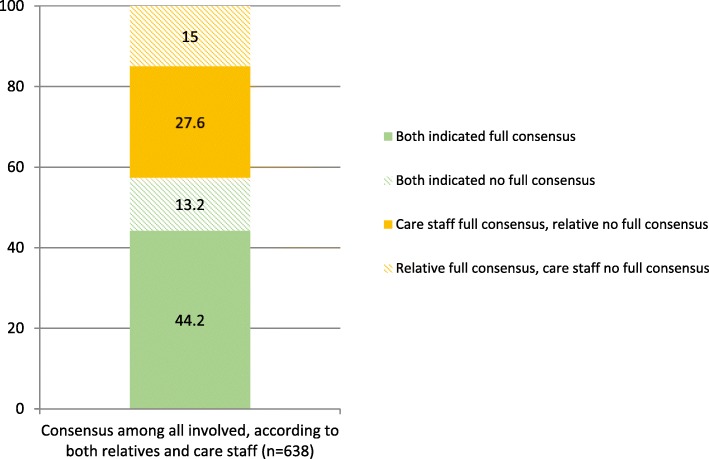
Comparing degree of consensus amongst all involved, according to care staff and relatives involved with the same resident

For 638 residents both the relative and care staff indicated the degree of consensus amongst all involved (see Fig. 3). Relatives and care staff both indicated full consensus was reached in 44.2% of residents and in 13.2% of residents both indicated no full consensus was reached amongst all involved. In over one of four cases (27.6%), full consensus was indicated by care staff, but not by relatives, while in 15% of cases this was the other way around.

### Factors associated with full consensus according to relatives

Table 2 shows associations between full consensus in treatment decisions among all involved according to relatives and country and patient, care and relative characteristics. In univariable analyses significant associations were found between full consensus and: country, resident’s comfort level, diagnosis of dementia, existence of multidisciplinary meetings, relation to the resident, a care provider explained palliative care to the relative, relative feeling fully involved in decision making and a higher level of family-physician communication. The multivariable model documented that variables associated with full consensus among all involved were: living in Poland (OR 2.07 [95% CI 1.10–3.89]) and Belgium (2.21 [1.27–3.84]) (compared to Finland), scoring a higher level of comfort in the last week of the resident’s life (1.66 [1.16–2.36]), the fact that resident did talk with them or someone else about preferred medical treatments (1.69 [1.10–2.61]), their relationship to the resident was other than child (2.08 [1.02–4.24]) (compared to spouse/partner), the receipt of an explanation of palliative care by a care provider (1.98 [1.33–2.96]), and a better rating of the family-physician communication (3.24 [2.28–4.60]). No interaction with country was observed.

**Table 2:** Patient, care and relative characteristics related to full consensus among all involved, according to relatives, univariable and multivariable analyses

	Full Consensus *N* = 457 (57.8%)	No full consensus *N* = 333 (42.2%))	Univariable OR (95% CI)	*p*-value	Multivariable OR (95% CI)	*p*-value
	N (%)	N (%)				
Country						
Finland (ref)^	59 (40.7)	86 (59.3)				
England	17 (68.0)	8 (32.0)	**3.18 (1.18–8.52)**	**.022**	1.87 (0.54–6.52)	.328
Italy	63 (64.3)	35 (35.7)	**2.49 (1.18–5.27)**	**.017**	2.05 (0.99–4.24)	.052
The Netherlands	106 (57.9)	77 (42.1)	**1.94 (1.21–3.11)**	**.006**	1.06 (0.56–1.99)	.083
Poland	84 (63.6)	48 (36.4)	**2.46 (1.53–3.97)**	**<.001**	**2.07 (1.10–3.89)**	**.025**
Belgium	128 (61.8)	79 (38.2)	**2.32 (1.53–3.52)**	**<.001**	**2.21 (1.27–3.84)**	**.005**
Patient characteristics						
Resident’s health in last week of life^a^						
≤ median (10)	232 (59.3)	159 (40.7)				
> Median (10)	202 (55.5)	162 (44.5)	0.87 (0.69–1.10)	.252		
EOLD-CAD Resident’s comfort in the last week of life^b^						
≤ mean (30)	203 (52.9)	181 (47.1)				
> mean (30)	197 (61.8)	122 (38.2)	**1.50 (1.11–2.02)**	**.008**	**1.66 (1.16–2.36)**	**.005**
Dementia						
no	211 (61.7)	131 (38.3)				
yes	228 (53.8)	196 (46.2)	**0.73 (0.55–0.98)**	**.037**		
Length of stay						
< 1 year	168 (57.5)	124 (42.5)				
≥ 1 year	265 (59.0)	184 (41.0)	1.05 (0.78–1.42)	.756		
Resident talked with relative or someone else about preferred medical treatment						
no or don’t know	331 (56.4)	256 (43.6)				
yes	124 (62.0)	76 (38.0)	1.32 (0.94–1.86)	.110	**1.69 (1.10–2.61)**	**.017**
Care facility characteristics						
Organization of multidisciplinary meetings in facility						
no or don’t know	61 (46.9)	69 (53.1)				
yes	375 (60.9)	241 (39.1)	**1.74 (1.15–2.62)**	**.009**		
No. care staff/10. occupied beds						
≤ 5	225 (62.7)	134 (37.3)				
> 5	204 (55.0)	167 (45.0)	0.75 (0.54–1.05)	.095		
Relative characteristics						
Relationship to resident						
Spouse/partner (ref.)	41 (46.1)	48 (53.9)				
Son/daughter	290 (57.2)	217 (42.8)	1.54 (.98–2.42)	.063	1.36 (.71–2.61)	.358
Other (ref)	123 (64.7)	67 (35.3)	**2.07 (1.23–3.49)**	**.006**	**2.08 (1.02–4.24)**	**.044**
Care provider explained what palliative care means						
no	122 (40.5)	179 (59.5)				
yes	328 (68.8)	149 (31.2)	**3.13 (2.32–4.23)**	**<.001**	**1.98 (1.33–2.96)**	**.001**
Relative did not really understand resident’s condition						
disagree	366 (58.5)	260 (41.5)				
agree	71 (55.0)	58 (45.0)	0.85 (0.56–1.29)	.435		
Relative expected resident would die, one month before death						
no or don’t know	257 (56.7)	196 (43.3)				
yes	196 (59.2)	135 (40.8)	1.07 (0.80–1.44)	.644		
Relative felt fully involved in all decision making						
disagree	37 (30.6)	84 (69.4)				
agree	404 (63.4)	233 (36.6)	**3.81 (2.56–5.68)**	**<.001**		
Family Perception of Physician-Family Communication (FPPFC)						
< mean (2)	116 (38.7)	184 (61.3)				
≥ mean (2)	289 (73.5)	104 (26.5)	**4.32 (3.22–5.80)**	**<.001**	**3.24 (2.28–4.60)**	**<.001**

^Finland is the reference category because the proportions of full consensus were lowest in this country

*Missing values: health = 35, comfort/symptom burden = 87, dementia = 24, length of stay = 49, resident talked about preferred treatment = 3, multidisciplinary meeting = 44, care staff/occupied beds = 60, relation = 4, care provider explained palliative care = 12, relative didn’t understand condition = 35, relative expected death = 6, relative felt fully involved = 32, FPPFC = 97

Bold printed OR and *p*-values are below the significance level of .05

^a^Higher scores indicate better health

^b^Higher scores indicate more comfort and less symptom burden

### Factors associated with full consensus according to care staff

Table 3 shows associations between full consensus on care and treatment in the last month of life, among all involved in care, according to care staff and country and patient, care and relative characteristics. Univariable analyses showed significant associations between full consensus and country, resident’s comfort level and the number of care staff per occupied beds. The multivariable model showed care staff were more likely to indicate full consensus if they lived in Italy (2.42 [1.41–4.15]), the Netherlands (2.21 [1.42–3.44]), Poland (3.47 [2.06–5.84]), or England (3.68 [1.81–7.47]) (compared to Finland) or if they reported a higher level of comfort in the last week of the resident’s life (1.57 [1.19–2.06]). No interaction with country was observed.

**Table 3:** Patient and care characteristics related to full consensus among all involved, according to care staff, univariable and multivariable analyses

	Full Consensus	No full consensus				
	*N* = 937 (73%)	*N* = 347 (27%)	Univariable OR (95% CI)	*p*-value	Multivariable OR (95% CI)	*p*-value
Country						
Finland (ref)^	153 (59.5)	104 (40.5)				
Belgium	168 (65.1)	90 (34.9)	1.22 (0.82–1.83)	.332	1.14 (0.75–1.72)	.538
Italy	146 (76.8)	44 (23.2)	**1.92 (1.13–3.28)**	**.016**	**2.42 (1.41–4.15)**	**.001**
The Netherlands	165 (77.5)	48 (22.5)	**2.29 (1.50–3.50)**	**<.001**	**2.21 (1.42–3.44)**	**<.001**
Poland	237 (82.6)	50 (17.4)	**3.07 (1.84–5.11)**	**<.001**	**3.47 (2.06–5.84)**	**<.001**
England	68 (86.1)	11 (13.9)	**4.02 (2.03–7.96)**	**<.001**	**3.68 (1.81–7.47)**	**<.001**
Patient characteristics						
Resident’s health in last week of life^a^						
≤ median (20)	516 (73.8)	183 (26.2)				
> median (20)	392 (73.4)	142 (26.6)	0.94 (0.71–1.24)	.657		
Resident’s comfort in the last week of life^b^						
≤ mean (30)	381 (69.0)	171 (31.0)				
> mean (30)	515 (77.4)	150 (22.6)	**1.49 (1.15–1.93)**	**.002**	**1.57 (1.19–2.06)**	**.001**
Dementia						
no	288 (76.6)	88 (23.4)				
yes	641 (71.4)	257 (28.6)	0.78 (0.59–1.04)	.091		
Length of stay						
< 1 year	388 (74.6)	132 (25.4)				
≥ 1 year	504 (72.1)	195 (27.9)	0.98 (0.75–1.27)	.862		
Resident expressed preferences about treatment in the last phase of life						
no or don’t know	728 (73.0)	269 (27.0)				
yes	199 (72.9)	74 (27.1)	1.06 (0.78–1.44)	.715		
Care facility characteristics						
Organization of multidisciplinary meetings in facility						
no or don’t know	208 (72.7)	78 (27.3)				
yes	696 (73.4)	252 (26.6)	1.10 (0.76–1.58)	.611		
No. care staff/10. occupied beds						
≤ 5	461 (77.0)	138 (23.0)				
> 5	423 (69.1)	189 (30.9)	**0.67 (0.48–0.93)**	**.016**		

^Finland is the reference category because the proportions of full consensus were lowest in this country

*Missing values: health = 51, comfort/symptom burden = 67, dementia = 10, length of stay = 65, resident expressed treatment preferences = 14, multidisciplinary meeting = 50, care staff/occupied beds = 73

Bold printed OR and *p*-values are below the significance level of .05

^a^Higher scores indicate better health

^b^Higher scores indicate more comfort and less symptom burden

## Discussion

This study showed that on an aggregated level, relatives indicated full consensus among all involved in treatment decisions in more than half of the residents in all countries, except in Finland. Care staff reported that on care and treatment in the last month of life full consensus was reached among all involved in care, in over half of the residents in Belgium and Finland and in over three quarters of residents in the other countries. When relatives and care staff reported about the same resident, care staff indicated full consensus more often than relatives. Relatives more often indicated full consensus when: they rated a higher level of comfort of the resident, the resident talked with them or someone else about preferred medical treatments, a care provider explained what palliative care is, they indicated better family-physician communication, their relation to the resident was other than child (compared to spouse/partner), or if they lived in Poland or Belgium (compared to Finland). Care staff more often indicated full consensus when they rated a higher level of comfort of the resident, or if they lived in Italy, the Netherland, Poland or England (compared to Finland).

### Full consensus: care staff vs. relatives

Both on an aggregated level and on resident level, care staff indicated full consensus more often and no consensus less often than relatives, which is probably due to their different perspectives. It is possible that care staff did not have a complete appreciation of all the relatives involved in the decision making and they might not have been aware when consensus was not reached among relatives themselves. While families feel that healthcare providers often ask for one family member to be the spokesperson and decision maker, most of the time this does not happen [30] and family members often prefer to make a decision together [11, 31]. However, family conflict in end-of-life decisions can arise, for instance in case of prior family conflict, when one family member asserts control in the decision making or when family members have difficulty communicating with each other [32, 33]. It is also possible that relatives set higher norms than care staff as regards when full consensus is reached. Since care staff encounter end-of-life events in their daily practice, they might not consider decisions they perceive as referring to smaller issues in their evaluation of consensus regarding treatments and care.

Additionally, it should be noted that the question for care staff included reference to a specific time frame, ‘the last month of life’, which was not included in the question for relatives. It is possible that relatives considered decisions that had been made much earlier, possibly when the resident had only been living in the care facility for a little while. At that time relatives and residents were probably still getting used to the new status quo and perhaps were not yet fully informed about options for treatment and care, which could have resulted in a lack of consensus. While during the last month of life, most residents had been living in the care facility for quite some time. Thus everyone involved had had time to adjust to the situation and to be informed about possible options, which could have resulted in full consensus in more cases. Therefore the possible difference in time frame could also have contributed to the differences in consensus as indicated by relatives and care staff.

### Differences between countries

Some difference between countries was found in the rate of full consensus, which was lower in Finland compared to the other countries. Previous research in long-term care in Finland found little opportunity for relatives to participate in decision making [34, 35], which could have played a role in these findings. However, socially desirable answers could play a role in indicating the degree of consensus, when individuals experience a strong norm regarding full consensus. This norm could be more pronounced in some countries and therefore differences between countries might be overestimated in this study.

### Importance of advance care planning, comfort care and good communication for indicating full consensus among all involved

The results indicate that advance care planning at a time when the resident was still able to express wishes could play a role in making the decision process run more smoothly, since relatives more often indicated full consensus when they or someone else was aware about the resident’s wishes for care. Advance care planning has been found to increase patients’ and families’ satisfaction with care [36], which might relate to attuning of care decisions. Families use their knowledge of patient preferences to make decisions [2, 11] and lack of such knowledge can complicate the decision making process [11] which relatives then perceive as burdensome [37]. Care providers also tend to emphasise the wishes of the patient when making decisions in palliative care [5]. When a patient’s wishes are known, it is probably easier to come to agreement.

Additionally, both care staff and relatives were more likely to indicate full consensus if they reported a higher comfort level of the resident, indicating good comfort care could make it easier for families to accept changes in care goals. Previous research has shown that when family caregivers have difficulty evaluating the patient’s quality of life, they can experience internal conflict which complicates the decision making process [38]. However, the association between comfort level and consensus could also be reversed, for instance if relatives or staff did not agree with a certain decision that was made, they could look back on the resident’s comfort level more critically and in hindsight report lower comfort. Another possibility is that lack of consensus could have caused a delay in treatment and care which resulted in lower comfort levels.

Good communication between relatives and care professionals also seems to influence the level of agreement as perceived by relatives, since relatives who indicated better family-physician communication (regarding the resident’s condition and care) and who were informed about palliative care more often indicated full consensus. When family’s acceptance and understanding of the patient’s condition does not match the actual situation, family may have unrealistic expectations, making it difficult to establish realistic treatment plans [39]. Lack of communication between relatives and care professionals, such as physicians, could contribute to relatives having such unrealistic expectations about care and make it hard to reach a consensus on care and treatment.

### Strengths and limitations

Among the strengths of this study are the inclusion of a large sample of representative LTCFs in six EU countries, and the use of different proxy responders which allowed the comparison of the perspectives of relatives and care staff. Retrospective data collection is deemed appropriate to collect data on end-of-life care and for this purpose it is more inclusive than prospective studies [40]. This study also has some limitations, for instance recall bias cannot be excluded. However, this was limited by including only residents who died in the last three months before participation in the study. Also it is possible that the sample of relatives who responded is not fully representative, since the absence of full consensus between relatives and staff could contribute to relatives not wanting to participate and fill-in the questionnaire [41]. Furthermore, this study does not identify the decision(s) based on which full consensus was or was not reached and when full consensus was not reached, and in the PACE study, we did not specify the parties involved in the decision making. Finally, while grouping of no consensus and consensus on major issues had to be undertaken to prevent non-convergence in GEE analyses, this did make our results less detailed and we cannot infer anything about association patterns across these answer categories.

## Conclusions

In most countries the frequency of full consensus among all involved in decisions on treatment and care was relatively high. Across countries care staff indicated full consensus more often and no consensus less often than relatives. This study indicates that advance care planning, comfort and good communication between relatives and care professionals could play a crucial role in achieving full consensus. Even in the absence of established palliative care services, a constant effort should be made to ensure that these elements of care and communication are of the highest standard.

## Additional file

Additional file 1: **Table S1.** Resident, care and relative characteristics included as independent variables **(**Microsoft Word document). Table provides rationale for factors included as independent variables possibly associated with indicating full consensus. (DOCX 32 kb)

## Data Availability

The datasets used and/or analysed during the current study are available from the corresponding author on reasonable request.

## Abbreviations

CI: Confidence interval
DEOLD study: Dutch End Of Life in Dementia study
EOLD-CAD: End-of-Life in Dementia – Comfort Assessment in Dying
EORTC: European Organisation for Research and Treatment of Cancer
EU: European Union
FPPFC scale: Family Perception of Physician-Family Communication scale
GEE: Generalized Estimating Equations
LTCF: Long-term care facility
OR: Odds ratio
PACE project: ‘Palliative Care for Older People’ project
SPSS: Statistical Package for the Social Sciences
USA: United States of America
