# Supplementary material for: Consensus on treatment for residents in long-term care facilities: perspectives from relatives and care staff in the PACE cross-sectional study in 6 European countries
Source: BMC Palliat Care. 2019 Aug 29;18:73. doi: 10.1186/s12904-019-0459-9 (PMC6714096; doi:10.1186/s12904-019-0459-9)
Supplement: Supplementary file 1 — Table S1. Resident, care and relative characteristics included as independent variables (Microsoft Word document). Table provides rationale for factors included as independent variables possibly associated with indicating full consensus. (DOCX 32 kb) [file 12904_2019_459_MOESM1_ESM.docx]

**Table 1: Resident, care and relative characteristics included as independent variables.**

| **Variable** | **Reference/Justification** |
| --- | --- |
| Family Perception of Physician-Family Communication (FPPFC) scale | Quality of the relationship with healthcare providers plays a role for family caregivers in making end-of-life care decisions [36].  To make a decisions in the patient’s best interest, surrogate decision makers often gather information from the clinicians [7]. |
| Resident’s health in last week of life Resident’s comfort during last week of life | The relative’s quality of life plays a role when family caregivers make treatment decisions [1, 32].  Lack of alignment in treatment goals between physician and family often occurred when patients had unstable/declining functional or cognitive health [16].  Surrogate decision makers view the patients’ best interest in terms of their quality of life [7]. |
| Relation to the resident | Nursing home physicians are more likely to involve close family members in decision making [1].  Staff and families are less likely to have similar perspectives on patient’s symptom burden when relatives are spouses or other relatives, compared with children [13]. |
| Care provider explained what palliative care means | Understanding of care (options) plays a role in decision making [1].  Care providers teach patients and families about prognosis and treatment options, to help shift them towards palliative treatment decisions [37].  Families or patients difficulty in understanding the limitations and complications of life-sustaining treatments was an important barrier to care goal discussions [38]. |
| Relative did not really understand resident’s condition | A shared understanding of the medical situation facilitates decisions on treatment and care [6].  Care providers change patients’ and families’ understanding of the patient’s condition to one that is consistent with the provider [37]. |
| Dementia | Lack of alignment in treatment goals between physician and family often occurred when patients had unstable/declining functional or cognitive health [16].  Medical staff considered family wishes more important to the decision-making process when the resident was cognitively impaired [4].  Patient’s incapacity to make care goal decisions was an important barrier to care goal discussions [38]. |
| Relative expected resident would die, one month before death | Care providers help patient and family understand the patient might not survive, to nudge them towards choosing palliative care [37]. |
| Relative felt fully involved in all decision making | The degree to which families participate in the decision making process may influence the goal-setting process [39]. |
| Resident talked with relative or someone else about preferred medical treatment/expressed preferences about treatment in last phase of life | Surrogate decision makers base their decisions on statements of preference by the patient [7]. |
| Relative felt fully involved in all decision making | Family satisfaction with treatment decisions was higher when medical staff considered family wishes to be important to decision-making [4]. |
| -No. care staff / 10. occupied beds  -Multidisciplinary meetings  -Length of stay | Keeping staff on the same page regarding care decisions was more difficult/impossible when:  -the resident-to-staff ration was high  -staff did not know the physician preferences for type and amount of communication  -the nursing assistant did not know the residents [6] |
